# Sensing of electrolytes in urine using a miniaturized paper-based device

**DOI:** 10.1038/s41598-020-70456-6

**Published:** 2020-08-12

**Authors:** Fariba Ghaderinezhad, Hatice Ceylan Koydemir, Derek Tseng, Doruk Karinca, Kyle Liang, Aydogan Ozcan, Savas Tasoglu

**Affiliations:** 1 Department of Mechanical Engineering, University of Connecticut, Storrs, CT 06269 USA; 2 Electrical and Computer Engineering, University of California, Los Angeles, CA 90095 USA; 3 Bioengineering, University of California, Los Angeles, CA 90095 USA; 4 California NanoSystems Institute, University of California, Los Angeles, CA 90095 USA; 5 Computer Science, University of California, Los Angeles, CA 90095 USA; 6 Department of Mechanical Engineering, Koc University, Sariyer, Istanbul, 34450 Turkey; 7 Koç University Arçelik Research Center for Creative Industries (KUAR), Koç University, Sariyer, Istanbul, 34450 Turkey; 8 Boğaziçi Institute of Biomedical Engineering, Boğaziçi University, Çengelköy, Istanbul, 34684 Turkey; 9 Koc University Research Center for Translational Medicine, Koç University, Sariyer, Istanbul, 34450 Turkey

**Keywords:** Biotechnology, Assay systems, Biomedical engineering, Imaging, Sensors and probes

## Abstract

Analyzing electrolytes in urine, such as sodium, potassium, calcium, chloride, and nitrite, has significant diagnostic value in detecting various conditions, such as kidney disorder, urinary stone disease, urinary tract infection, and cystic fibrosis. Ideally, by regularly monitoring these ions with the convenience of dipsticks and portable tools, such as cellphones, informed decision making is possible to control the consumption of these ions. Here, we report a paper-based sensor for measuring the concentration of sodium, potassium, calcium, chloride, and nitrite in urine, accurately quantified using a smartphone-enabled platform. By testing the device with both Tris buffer and artificial urine containing a wide range of electrolyte concentrations, we demonstrate that the proposed device can be used for detecting potassium, calcium, chloride, and nitrite within the whole physiological range of concentrations, and for binary quantification of sodium concentration.

## Introduction

Deviation of inorganic ion concentrations, such as sodium (Na^+^), potassium (K^+^), calcium (Ca^2+^), and chloride (Cl^−^), from the commonly accepted healthy ranges and the presence of nitrite (${\text{NO}}_{2}^{-}$) in human body fluids can be a symptom of a disorder or a dysfunction of an organ^1^. If such a malfunction goes undiagnosed and left untreated, it can lead to serious health issues. Regularly monitoring sodium, potassium, and calcium levels in urinary excretion can help to diagnose various disorders such as hypertension and cardiovascular diseases, hyperkalemia or hypokalemia (disorders which can be caused by changes in potassium intake), kidney disease or injury, adrenal gland problems, rickets, hypothyroidism, steatorrhea, vitamin D overdose, and renal tubular acidosis^2–6^. Additionally, measuring these cations is useful for people with urinary stone disease since calcium is the main component of urinary stones; urinary sodium and potassium are also related to urinary calcium excretion^7^. Moreover, sodium and potassium intake is commonly analyzed via urine samples^4^. Chloride ion measurement is used in the diagnosis of disorders such as cystic fibrosis and diabetic acidosis^8^, while nitrite is also used to estimate the probability of a urinary tract infection (UTI) since nitrite is a metabolic product of typical pathogens of the urinary tract^9^. In healthy urine, nitrite is not present, but rather is produced by bacterial reduction of urinary nitrate^10^.

For determining the cation and anion concentrations in urine, different analytical methods are used, including flame emission spectrophotometry^11^, ion selective electrodes^12^, ion chromatography^13^ and capillary electrophoresis^14^, all of which are accurate and precise. However, the total analysis time, the amount of sample required, and the analysis cost of these methods may be relatively high^1,11,15,16^. Alternatively, paper-based devices are cost-effective, provide rapid analysis, and have a wide-range of applications in many fields^17^, including pharmaceutics^18^, food safety^19,20^, environmental monitoring^21,22^ and medical diagnostics^23–28^. Dipsticks and lateral flow immunoassays (LFIA) are just two examples of common paper-based diagnostic devices with many real-world applications. Nevertheless, paper-based devices which measure clinically-important analytes are still scarce^24^; there is an unmet need for developing such devices in a high-throughput, low-cost manner.

**Figure 1:**
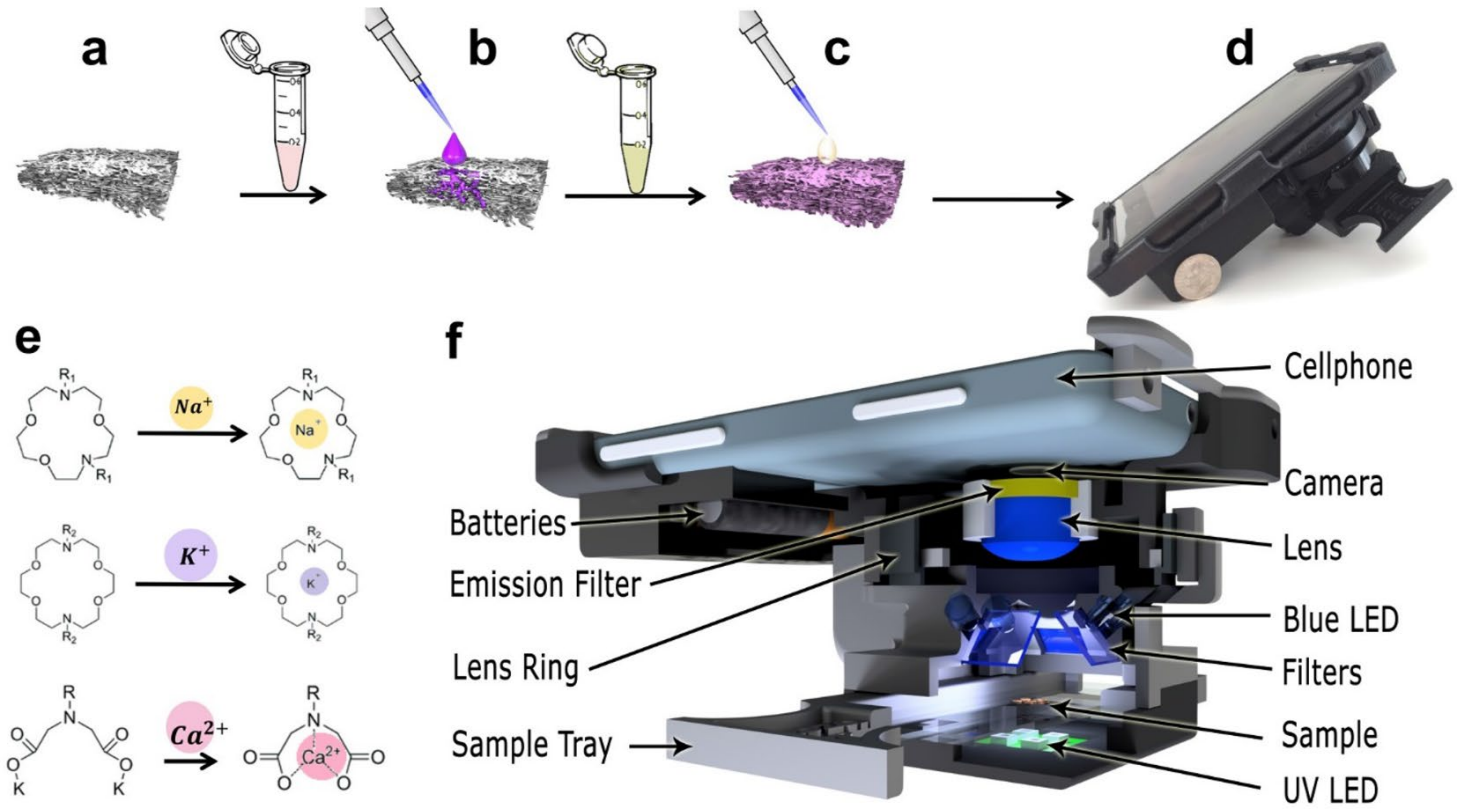
Schematic procedure of measuring the concentration of cations. (**a**) Chromatography paper cut with laser cutter (Ø = 0.25 in). (**b**) Depositing 2 μl of fluorescent probe (Sodium green, PBFI, and Fluozin) on paper. (**c**) Adding the solution that contains the Na^+^, K^+^, or Ca^2+^ ions. (**d**) Reading the fluorescent intensity by a smartphone-enabled platform. (**e**) Chelation mechanism of fluorescent probes (from top to bottom: sodium green, PBFI, and Fluozin). (**f**) Illustration of the portable smartphone-based reader.

Regarding the importance of measuring electrolytes in urine, finding a cost-effective, sensitive, and simple-to-use device can be valuable in both resource-limited regions and developed countries. To address this need, we developed a cost- and time-effective paper-based method for measuring the concentration of Na^+^, K^+^, Ca^2+^, Cl^−^, and ${\text{NO}}_{2}^{-}$ ions in urine (Fig. 1). For this purpose, we chose a fluorescent probe for detecting Na^+^, K^+^, and Ca^2+^ ions and a colorimetric method for detecting Cl^−^ and ${\text{NO}}_{2}^{-}$ ions. We added the reagents on a paper matrix and deposited the sample, followed by reading the fluorescent intensity and/or color intensity for quantification of the corresponding target ion concentrations. Our results show that Na^+^, K^+^, and Ca^2+^ in their physiological concentrations, Cl^−^ concentrations from 50 to 300 mM, and ${\text{NO}}_{2}^{-}$ concentrations from 0.05 to 2 mM can be accurately detected in artificial urine.

## Materials and equipment

Sodium Green (S6900) and Tetraammonium Salt, PBFI (P1265MP), Tripotassium Salt, FluoZin-1 (F24180); Corning 96-Well Clear Bottom Black Polystyrene Microplate (07-200-565) from Thermo Fisher Scientific Inc., MA, US; Lactic acid (W261106), Citric acid (C0759), Magnesium sulphate (746,452), Ammonium chloride (A9434), Calcium chloride (C1016), Potassium chloride (746,436), Sodium chloride (S9888), Sodium bicarbonate (792,519), Manganese (II) chloride (244,586), Tris base (648,310), Tris hydrochloride (10,812,846,001), Nickel (II) chloride, Sodium nitrite (237,213), Sulfanilamide (S9251N), N-(1-Naphthyl)ethylenediamine (33,461), and Chloride assay kit (MAK023) from Sigma, US; Potassium phosphate dibasic (7,758,114) from Acros Organics, Geel, Belgium; Potassium dihydrogen phosphate (104,873) and Urea (108,487) from EMD Milipore Co., MA, US; Dimethyl Sulfoxide, DMSO, (25-950-CQC), Corning Inc., NY, US; Whatman No. 1, GE healthcare life sciences, IL, US; Synergy H1 Hybrid reader, Biotek, VT, US; CO_2_ laser-cutter, Universal Laser Systems Inc., AZ, US; Blue LEDs (516-2800-1-ND), Digi-Key Corporation, MN, US; Ultraviolet (UV) LEDs (160-2184CT-ND), Digi-Key Corporation, MN, US; Excitation bandpass filter (ET470/40x), Chroma Inc., VT, US; Long-pass emission thin-film filter (FF01-500/LP-23.3-D), Semrock Inc., NY, US; Thermoplastic 3D printing filament (ABS P430 Plus) and 3D printer (Dimension Elite) from Stratasys Ltd., MN, US.

## Methods

**Figure 2:**
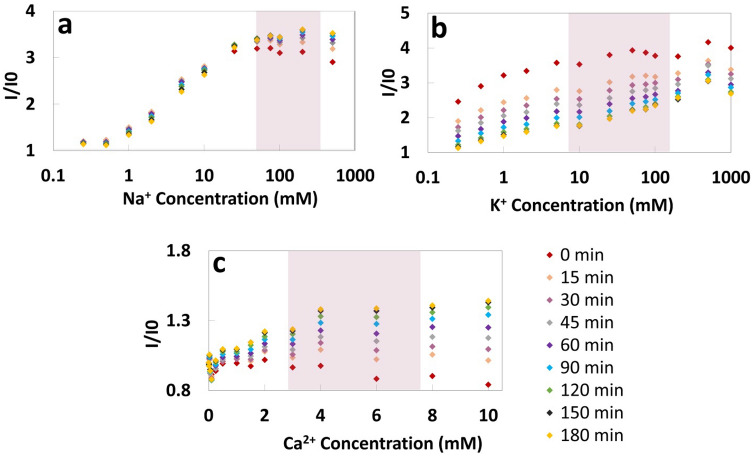
Investigating the effect of elapsed time on fluorescent intensity of probes for (**a**) Na^+^, (**b**) K^+^ and (**c**) Ca^2+^ ions for a range of concentrations. Elapsed time refers to the time passed between depositing the ion solution on the paper and reading the intensity: elapsed time varied from 0 to 3 h. Pink shaded area shows the physiological ion concentration range in human urine.

**Figure 3:**
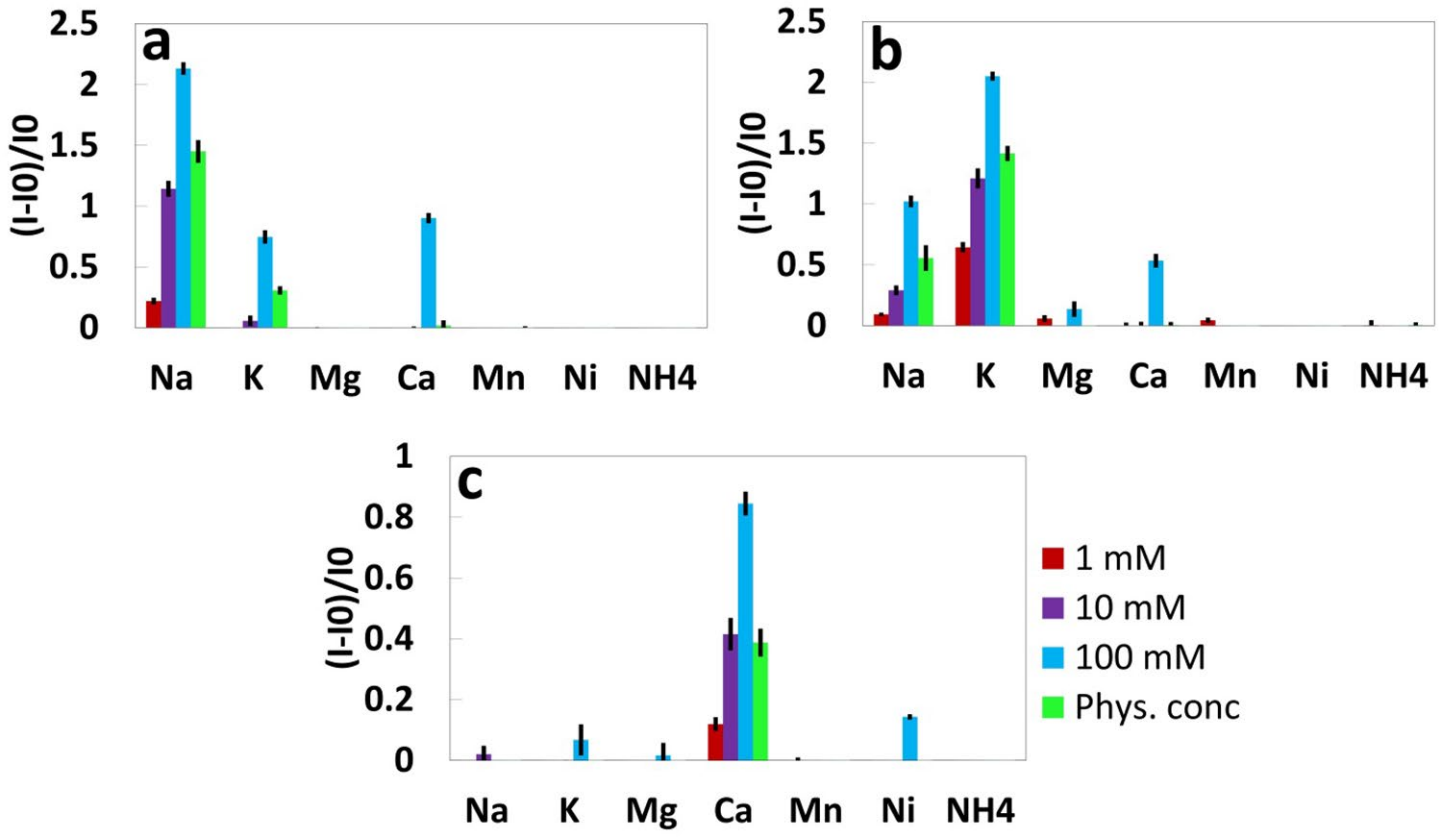
Selectivity of fluorescent probes. Selectivity of 25 μM (**a**) Sodium green, (**b**) PBFI, and (**c**) Fluozin towards different ions in Tris buffer solution (150 mM, pH 7.4) at concentrations of 1, 10, 100 mM, and their maximum physiological concentration. Excitation/Emission wavelength of Sodium Green (λ_ex_/λ_em_: 485 nm/541 nm), PBFI (λ_ex_/λ_em_: 360 nm/450 nm), and Fluozin (λ_ex_/λ_em_: 485 nm/541 nm). Error bars represent the standard error of the mean (n = 6).

**Figure 4:**
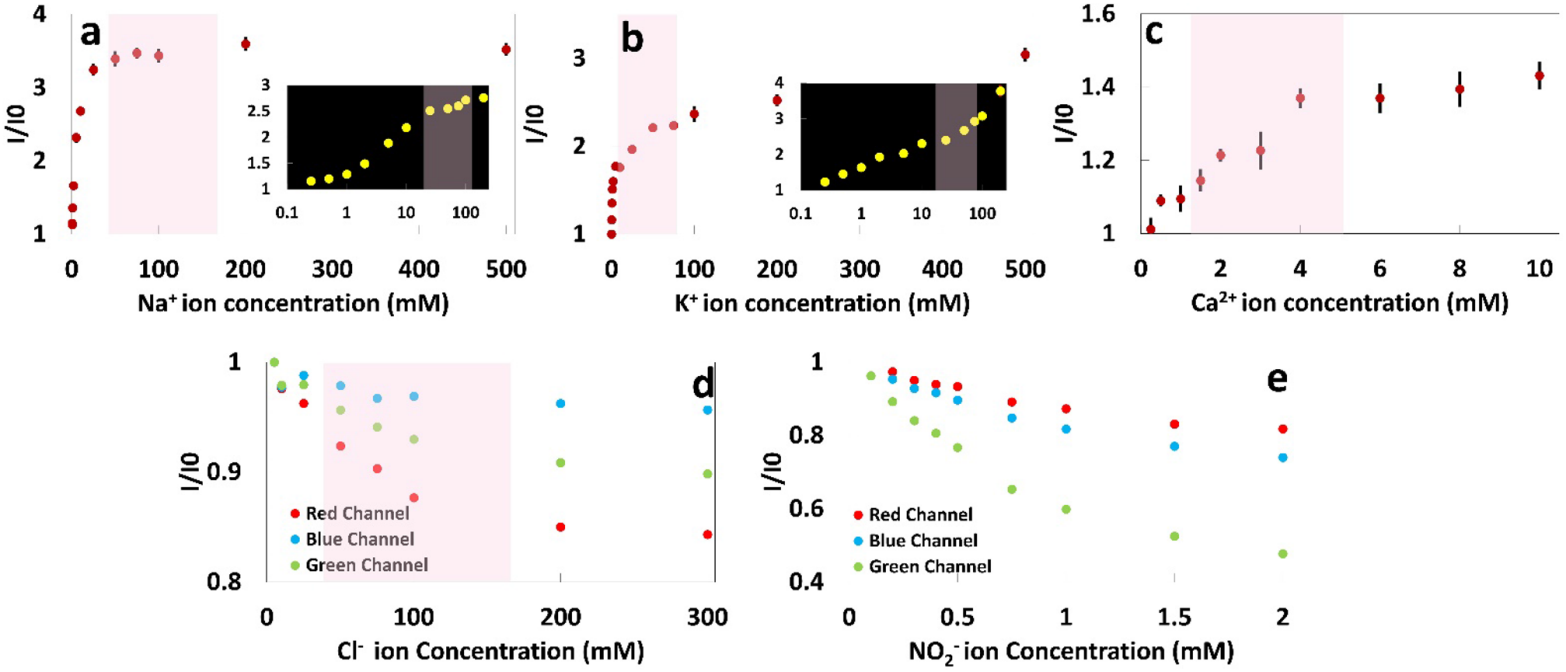
Na^+^, K^+^, Ca^2+^, Cl^−^, and ${\text{NO}}_{2}^{-}$ ion concentration measurements in Tris buffer (150 mM, pH 7.4) using fluorescent probes (Sodium Green, PBFI, and Fluozin, respectively) and the RGB model. Calibration curves of (**a**) Na^+^, (**b**) K^+^, and (**c**) Ca^2+^ ions on paper matrix at a constant probe concentration of 25 μM in DMSO (Sodium Green (λ_ex_/λ_em_: 485 nm/541 nm), PBFI (λ_ex_/λ_em_: 360 nm/450 nm), and Fluozin (λ_ex_/λ_em_: 485 nm/541 nm)). Red, Blue, and Green channels of the RGB model for different concentrations of (**d**) Cl^−^ and (**e**) ${\text{NO}}_{2}^{-}$ ions. Insets in (**a**) and (**b**) show the logarithmic scale of Na^+^ and K^+^ concentrations, respectively. Error bars represent standard error of the mean (n = 6). Pink shaded area shows the physiological ion concentration range in human urine.

**Figure 5:**
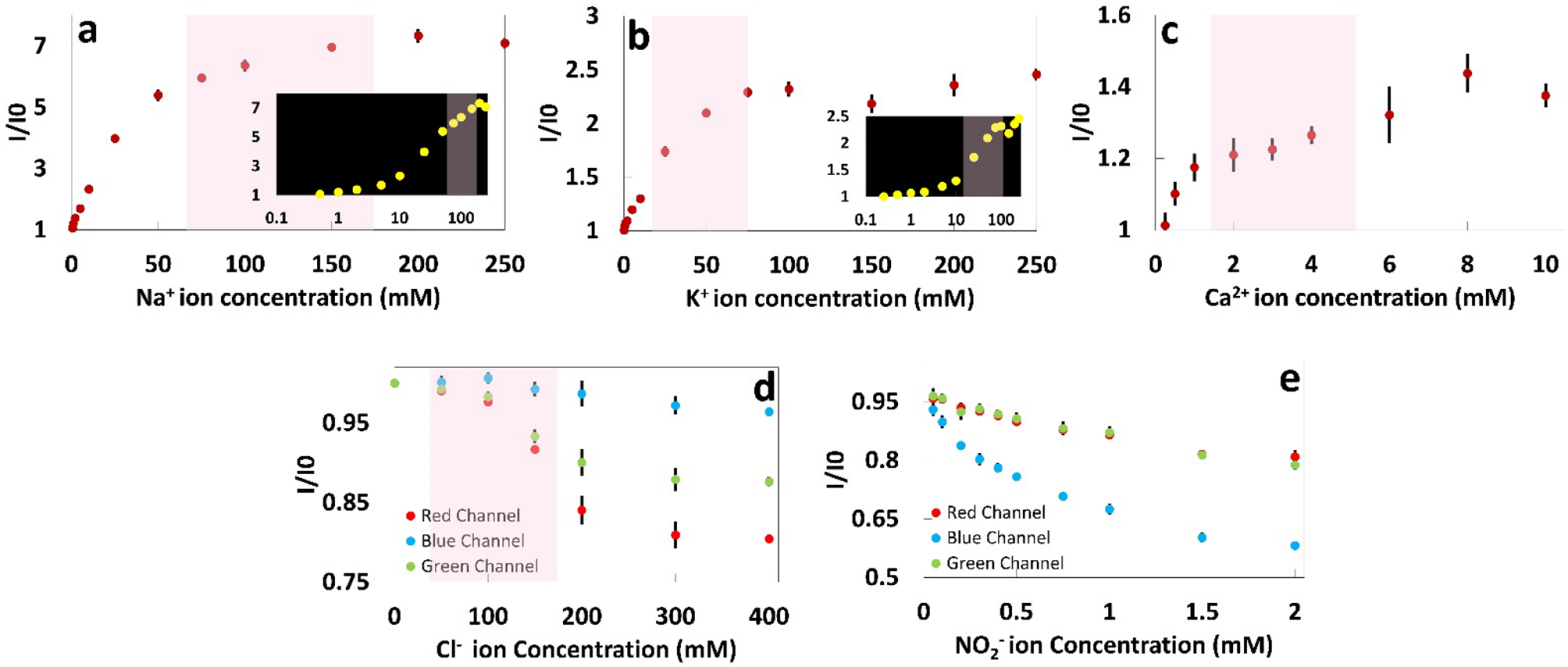
Na^+^, K^+^, Ca^2+^, Cl^−^, and ${\text{NO}}_{2}^{-}$ ion measurements in artificial urine (pH 6) using fluorescent probes (Sodium Green, PBFI, and Fluozin, respectively) and the RGB model. Calibration curves of (**a**) Na^+^, (**b**) K^+^, and (**c**) Ca^2+^ ions on paper matrix at a constant probe concentration of 25 μM in DMSO (Sodium Green (λ_ex_/λ_em_: 485 nm/541 nm), PBFI (λ_ex_/λ_em_: 360 nm/450 nm), and Fluozin (λ_ex_/λ_em_: 485 nm/541 nm)). Red, Blue, and Green channels of the RGB model for different concentrations of (**d**) Cl^−^ and (**e**) ${\text{NO}}_{2}^{-}$ ions. Insets in (**a**) and (**b**) show the logarithmic scale of Na^+^ and K^+^ concentrations, respectively. Error bars represent standard error of the mean (n = 6). Pink shaded area shows the physiological ion concentration range in human urine.

**Figure 6:**
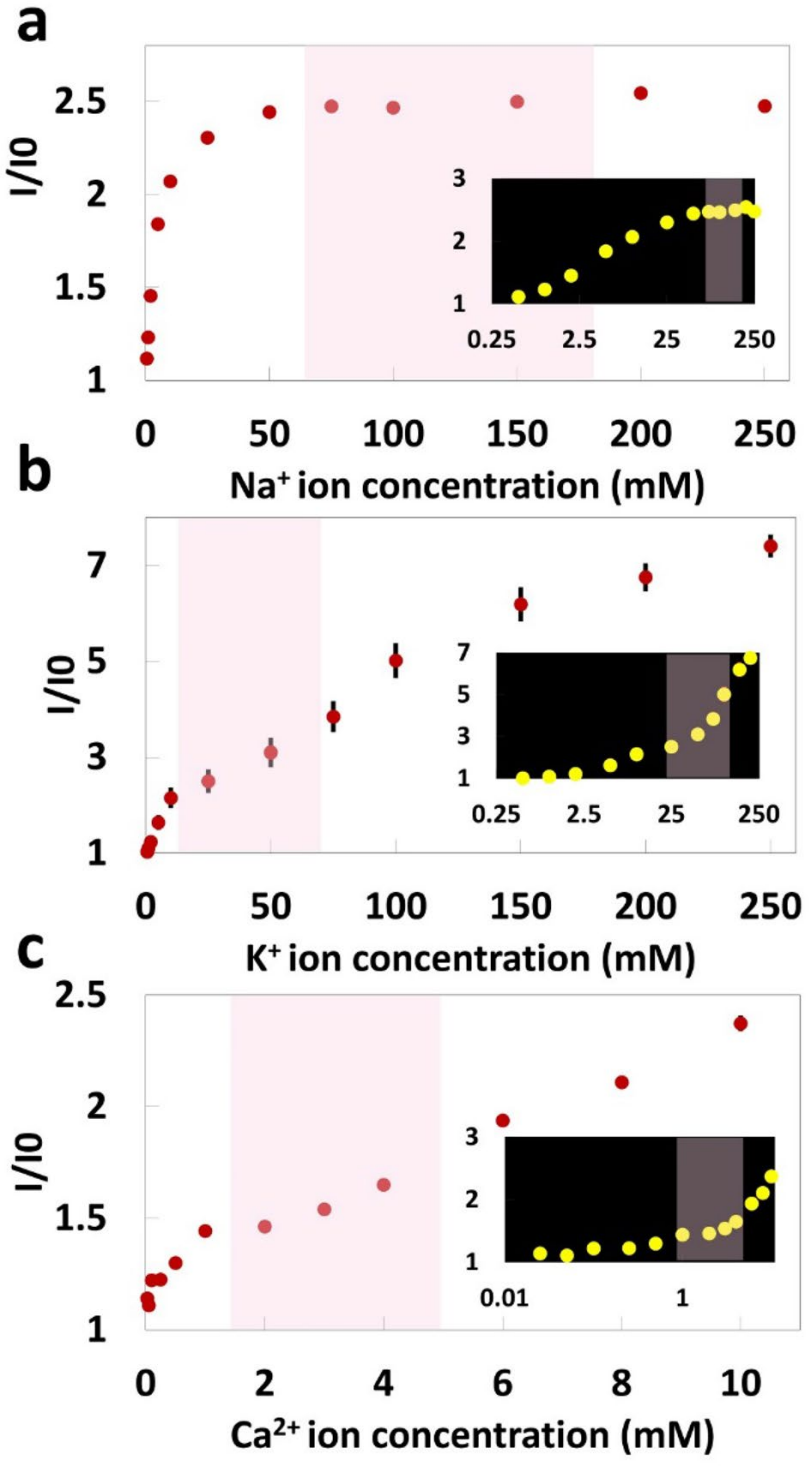
Na^+^, K^+^, and Ca^2+^ ion measurements in artificial urine (pH 6) using smartphone-enabled platform. Calibration curves of (**a**) Na^+^, (**b**) K^+^, and (**c**) Ca^2+^ ions on paper matrix at a constant probe concentration of 250 μM in DMSO (Sodium Green (λex/λem: 485 nm/541 nm), PBFI (λex/λem: 360 nm/450 nm), and Fluozin (λex/λem: 485 nm/541 nm)). Insets show the logarithmic scale of Na^+^, K^+^, and Ca^2+^ concentrations, respectively. Error bars represent standard error of the mean (n = 6). Pink shaded area shows the physiological ion concentration range in human urine.

To quantify the concentrations of Na^+^, K^+^, and Ca^2+^ ions, fluorescent probes Sodium Green as the sodium indicator, PBFI potassium-sensitive dye, and Fluozin calcium indicator were chosen. Chromatography paper (Whatman No. 1) was used as the reaction matrix on which the fluorescent probe solutions with concentrations of 25 μM were added. The fluorescent probes were diluted using DMSO since this organic solvent prevents the hydrolysis. During the first phase of our experiments, a microplate reader was used to measure the fluorescence intensities of the probes. To accommodate the microplate reader, the papers were cut into a round shape (Ø = 0.25 in) using a CO_2_ laser-cutter. 2 μl of fluorescent probe was deposited on the paper matrix followed by 2 μl of the metal ion solution. The papers were then placed at the bottom of the microwells in 96-well plates for reading the fluorescent intensity of the probes. The excitation/emission peaks were 485 nm/541 nm for detection of sodium and calcium ion and 360 nm/450 nm for detection of potassium ion. The effect of elapsed time on the fluorescent intensity after depositing the ion solution on the paper and before reading the intensity was investigated (Fig. 2). Additionally, the sensing selectivity of the sensors in the presence of other ions including magnesium, manganese, nickel, and ammonium, that are normally available in human urine, was studied (Fig. 3). Furthermore, the detection of ions was tested in two different solutions: Tris buffer (150 mM, pH 7.4) and artificial urine (pH 6) (Figs. 4a–c and 5a–c). To quantify Cl^−^ concentration in Distilled Water (DI) and artificial urine, 2 μl and 4 μl of the commercially available Cl^−^ reagent were spotted on paper, respectively. This reagent for Cl^−^ gives a colorimetric product, which has 620 nm and is proportional to the chloride present in the sample, from the reaction between 2,4,6-Tris(2-pyridyl)-s-triazine (TPTZ) and Fe^2+^. After drying the reagent, 2 μl of sample containing Cl^−^ ions was deposited on paper. The blue color on the paper was captured using the camera of an iPhone 6 and the images were processed by a custom-developed image processing algorithm. The results are shown in Fig. 4d (Cl^−^ ions in DI) and Fig. 5d (Cl^−^ ions in artificial urine). To detect ${\text{NO}}_{2}^{-}$ ions, we used a reagent containing sulfanilamide (50 mM), N-(1-Naphthyl)ethylenediamine (10 mM) and citric acid (330 mM)^29^. After depositing 2 µl of the reagent on the paper, 2 µl of sample containing different concentrations of nitrite ions was added. The color of the paper changed to pink upon adding the nitrite ion. This color change was captured by the camera of an iPhone 6 and the acquired images were processed by a MATLAB script. The quantified results are presented in Fig. 4e (${\text{NO}}_{2}^{-}$ in Tris buffer, 150 mM, pH 7.4) and Fig. 5e (${\text{NO}}_{2}^{-}$ ions in artificial urine). During the next phase of experiments, we replaced the plate reader with the smartphone-enabled platform and measured the concentration of cations in an artificial urine sample (Fig. 6). The screenshots of the app designed for this smartphone-enabled platform is shown in Figure S1 (Supplementary Information).

### Preparation of ion solutions

Sodium, potassium, calcium, and nitrite ion solutions in Tris buffer (150 mM, pH 7.4) and chloride ion in DI were prepared using sodium chloride, potassium chloride, calcium chloride, sodium nitrite, and sodium chloride, respectively. The stock solutions were serially diluted to the desired concentrations of sodium (0.25–250 mM), potassium (0.25–250 mM), calcium (0.25–10 mM), chloride (10–300 mM) and nitrite (0.1–2 mM) ions.

To investigate the selectivity of the fluorescent probes for detecting cations, different ion solutions were prepared at four concentrations including 1, 10, 100 mM, and a physiological concentration. For the physiological concentration, the maximum ion concentration found in human urine was considered, as follows: sodium (220 mM), potassium (125 mM), calcium (10 mM), magnesium (15 mM), manganese (1 mM), nickel (1 mM) and ammonium (70 mM). Sodium chloride, potassium chloride, calcium chloride, magnesium sulfate, manganese (II) chloride, nickel (II) chloride, and ammonium chloride were used for preparing these solutions, respectively. To investigate the selectivity of each fluorescent probe, 2 μl of different ion solutions were deposited on the paper after depositing 2 μl of the fluorescent probe solution. By reading the fluorescent intensity in the presence of each of the ions, the results were compared. The selectivity of the fluorescent probes was determined based on the intensity of each fluorescent probe in the presence of ions in the solution.

### Preparation of artificial urine and standard solutions

The artificial urine solution was prepared as reported by Martinez et al.^30^. This solution contained 1.1 mM lactic acid, 2.0 mM citric acid, 25 mM sodium bicarbonate, 170 mM urea, 2.5 mM calcium chloride, 100 mM sodium chloride, 2.0 mM magnesium sulfate, 10 mM sodium sulfate, 7.0 mM potassium dihydrogen phosphate, 7.0 mM dipotassium hydrogen phosphate, and 25 mM ammonium chloride dissolved in DI water. The pH of the solution was adjusted to 6 using 37% hydrochloric acid. To prepare artificial urine solutions containing different concentrations of sodium, potassium and chloride ions, sodium bicarbonate and sodium chloride with a molar ratio of 4, potassium dihydrogen phosphate and dipotassium hydrogen phosphate with a molar ratio of 1, and calcium chloride, ammonium chloride, and sodium chloride with a molar ratio of (1: 10: 40) were used, respectively.

### Design and fabrication of smartphone-enabled reader

A cost-effective and smartphone based reader was developed, which made up of a 3D-printed case, aligned with the camera of the smartphone, an external lens, an excitation filter, an emission filter, light emitting diodes (LEDs), a sample tray, and three AAA batteries (Fig. 1e). We used a smartphone (i.e., Nokia Lumia 1020) in the design of the mobile fluorescence reader, which enables acquisition of raw format (i.e. digital negative (DNG)) images (7,152 × 5,360 pixels) at an aspect ratio of 4:3. The complementary metal–oxide–semiconductor (CMOS) image sensor has a pixel size of 1.12 μm and the built-in 6- lens of the smartphone has a focal length (f) of 7.2 mm and a relative aperture of f/2.2. In addition, using the regular pro-camera application settings, we were able to adjust camera parameters (i.e. white balance, focus, ISO speed, exposure time, and contrast) to capture optimal images. We used eight blue LEDs to excite the fluorescent probes conjugated to sodium and calcium ions and an ultraviolet (UV) LED for potassium ion measurements. Three rechargeable batteries (1.5 V) were utilized to power all LEDs turning on 3 position rocker switch. The emission spectrum of the blue LEDs was filtered using a bandpass filter (470 nm/ 40 nm). An external lens with a focal length f = 30 mm was used to create a magnification factor of 0.24 (i.e. f/f2) between the sample plane and the CMOS sensor of the mobile-phone, which helped us achieve a large FOV (~ 1 cm^2^) at one shot. To manually adjust the focal plane, we placed a ring mount that allows user to adjust the distance between the external lens and the sample plane^31,32^. The fluorescence emissions of probes were collected using a long-pass filter with a cut-off wavelength of 515 nm. The custom-designed case of our fluorescence reader was 3D-printed using black thermoplastic material.

After capturing the fluorescence image, a custom-designed smartphone application wirelessly transmits the image to a server (which could also be a local computer or laptop) for rapid and automated quantification of the ion concentrations^33–36^. The images are processed using a custom-developed algorithm and the concentration measurement of each ion in the sample of interest is returned back to the smartphone within ~ 90 s, and is displayed to the user through the same smartphone application.

## Results and discussion

### Effect of elapsed time on fluorescent intensity of probes

We detected Na^+^, K^+^, and Ca^2+^ ions in Tris buffer solution (150 mM, pH 7.4) at different concentrations at room temperature and investigated the effect of elapsed time on the intensity of fluorescent probes during a 3-h interval. The results (Fig. 2) showed that each of ions behaved differently toward passing time while the mechanism for these different behaviors is not clear. The intensity of the fluorescent probe for detecting Na^+^ did not change significantly over time. But, the intensity of the fluorescent probe for detecting K^+^ decreased and it became almost constant after 2 h. In contrast to this change for K^+^ ion, the intensity of the fluorescent probe for detecting Ca^2+^ increased gradually and became constant after approximately 150 min. We adopted 120 min for Na^+^ and K^+^ and 150 min for Ca^2+^.

### Selectivity of fluorescent probes

Urine is comprised of mostly water, but it contains other substances, including ions of various concentrations. Magnesium (Mg^2+^), manganese (Mn^2+^), nickel (Ni^2+^) and ammonium (NH_4_^+^) are some of the most common ions in urine, in addition to sodium, potassium, and calcium ions. We studied the selectivity of the sodium, potassium, and calcium fluorescent probes (sodium green, PBFI, and Fluozin, respectively) in presence of Mg^2+^, Mn^2+^, Ni^2+^, and NH_4_^+^ ions. Figure 3a shows that for all of the concentrations (1, 10 and 100 mM) of these ions, the fluorescent probes performed as expected: sodium green had the highest fluorescent intensity in presence of Na^+^, PBFI had the highest fluorescent intensity in presence of K^+^, and Fluozin had the highest fluorescent intensity in presence of Ca^2+^.

To simulate conditions close to those of a real urine sample, we considered the maximum physiological concentration of each ion in urine and investigated the extent of interference. Sodium Green had the highest fluorescent intensity in the presence of Na^+^ (1.88- and 2.41-fold higher intensity ratio, i.e. I/I0, than that in the presence of K^+^ and Ca^2+^ ions, respectively). Moreover, PBFI showed the highest intensity in the presence of K^+^ (1.55- and 2.4-fold higher intensity ratio than that in the presence of Na^+^ and Ca^2+^ ions, respectively). Similarly, Fluozin showed the highest intensity in the presence of Ca^2+^ (1.77-, 1.59-, and 1.52-fold higher intensity ratio than that in the presence of Mg^2+^, Mn^2+^ and Ni^2+^ ions).

### Effect of the concentration of the electrolytes on signal intensity

The concentration of Na^+^, K^+^, Ca^2+^, and Cl^−^ in healthy urine is in the range of 100–260 mmol/day (65–175 mM), 25–100 mmol/day (15–70 mM), 2.5–7.5 mmol/day (1.5–5 mM), and 80–250 mmol/day (50–170 mM), respectively, while healthy urine should not contain any nitrite ion^37^ (Fig. 2). It should be noted that since the volume of urination per day varies between 0.8 and 2 L, we considered an approximate volume of 1.5 L for calculating the physiological concentration range of ions in mM. To cover the whole physiological range of urinary ion concentrations, we prepared solutions with a wide range of concentrations. It can be seen in Fig. 4a–c that the fluorescent intensity of probes increased with increasing concentration of ions in Tris buffer (150 mM, pH 7.4). The fluorescent intensity of the sodium and potassium fluorescent probes increased 351.9% and 304.3% by increasing the Na^+^ and K^+^ ion concentrations from zero to 500 mM, respectively. By increasing the concentration of Ca^2+^ in the buffer solution from zero to 10 mM, the intensity of the probe increased 143%. The fluorescent intensity ratio as a function of the concentration can be expressed as

1 $$\frac{I}{{I}_{0}} = A-B{e}^{-\propto C}$$

where $I$ and ${I}_{0}$ are the intensities of the solution with and without the target ions, respectively, $\propto$ is the saturation decay constant, C is the ion concentration in the solution, and A and B are constants^38^.

The saturation decay constant for Na^+^, K^+^, and Ca^2+^ ions in Tris buffer solution (150 mM, pH 7.4) and their R-squared values are summarized in Table 1. The high R-squared values obtained from the exponential curve fitting (Eq. 1) to the experimental data is representative of a good agreement between the experimental results and the theory. The results showed that at low ion concentrations, the intensity ratio was linearly proportional to the ion concentrations; this linearity occurred in the concentration range of 0–5 mM (R-squared 0.922), 0–1 mM (R-squared 0.958), and 0–4 mM (R-squared 0.955) for Na^+^, K^+^, and Ca^2+^ ions in Tris buffer (150 mM, pH 7.4), respectively. By increasing the concentration of ions, a deviation from linearity occurred which could be due to the absorption of the emitted signals by the ions in solution^39^.

**Table 1:** Calibration parameters and Limit of Detection (LOD) of sodium, potassium and calcium ions.

Media	Ion	Calibration curve							R-squared^c^	Precision
		Calibration range^a^ (mM)	Linear regression equation’s Constants^b^		R-squared^c^	Exponential equation’s constants^d^				LOD (mM)
			A	B		A	B	$\alpha$		
Tris buffer^f^ (150 mM, pH 7.4)	Na^+^	0–5	0.26	1.06	0.985	2.64	1.5	0.13	0.99	0.77
	K^+^	0–1	0.51	1.03	0.958	3.79	2.12	0.017	0.88	0.33
	Ca^2+^	0–4	0.086	1.01	0.955	1.45	0.46	0.31	0.97	0.99
Artificial urine^e^ (pH 6)	Na^+^	0–10	0.13	1.01	0.978	7.13	6.11	0.024	0.997	1.90
	K^+^	0–10	0.03	1.02	0.962	2.36	1.36	0.031	0.989	2.39
	Ca^2+^	0–2	0.11	1.01	0.854	1.43	0.39	0.256	0.922	1.13
Artificial urine^f^ (pH 6)	Na^+^	0–5	0.106	0.65	0.97	1.54	0.91	0.15	0.99	1.26
	K^+^	10–150	0.0198	1.16	0.99	–	–	–	–	0.85
	Ca^2+^	0.5–10	0.0166	0.19	0.99	–	–	–	–	1.2

^a^Actual linear range is wider.

^b^Linear Regression Equation is: $\frac{I}{{I}_{0}}=A\times C+B$, where A and B are constant and C is the concentration (mM).

^c^Correlation coefficient.

^d^Exponential Equation is $\frac{I}{{I}_{0}}=A-B{e}^{-\alpha C}$.

^e^A benchtop plate reader is used for measuring the concentration of ions.

^f^Smartphone-based device is used for measuring the concentration of ions at a constant probe concentration of 250 μM.

*Number of measurements for all the experiments is 6.

Limit of detection (LOD) for measuring the concentration of ions was calculated according to the following formula^40^:

2 $$LOD=3\left(\frac{{S}_{y}}{S}\right)$$

where ${S}_{y}$ is the standard deviation of the response of the linear curve and $S$ is the slope of the linear calibration curve. The LOD and calibration parameters for detecting Na^+^, K^+^ and Ca^2+^ ions in Tris buffer (150 mM, pH 7.4) are summarized in Table 1.

To quantify the concentration of chloride and nitrite ions, the RGB model, as one of the most common methods for color detection, was used. In RGB images, there are three color channels; Red, Green, and Blue. Figure 4d,e present the components of the RGB model for different concentrations of chloride and nitrite ions, respectively, to demonstrate that the intensity change mostly occurs in the red and blue channels. Therefore, these channels have the highest sensitivity; for example, the LOD of the nitrite ion using the blue channel was 0.239 mM.

### Detecting electrolytes in artificial urine

To simulate human urine, we synthesized an artificial urine solution with a wide range of ion concentrations. Figure 5 shows the results of detecting Na^+^, K^+^, Ca^2+^, Cl^−^, and ${\text{NO}}_{2}^{-}$ ions in artificial urine. The fluorescent intensity of the cations was measured using a plate reader. It can be seen that by increasing the concentration of cations, the corresponding fluorescent intensities increase. Using Eq. (1), the saturation decay ($\propto$) of Na^+^, K^+^, and Ca^2+^ ions in artificial urine was calculated. The saturation decay and R-squared values are summarized in Table 1. These results clearly demonstrate that Na^+^, K^+^, and Ca^2+^ ions in urine can be detected with high precision. Increasing the concentration of Na^+^ and K^+^ from 50 to 250 mM and from 5 to 150 mM within the physiological range led to a 31.4% and 82.3% increase in their fluorescent probe intensity, respectively. Moreover, by increasing the concentration of Ca^2+^ within the physiological range (1–10 mM), the fluorescent intensity of Fluozin increased 17.11%. The calibration parameters and limit of detection of Na^+^, K^+^, and Ca^2+^ are summarized in Table 1. Furthermore, Fig. 5d shows the results of detecting Cl^−^ in artificial urine using 4 μl of reagent following by adding 2 μl of sample containing the Cl^−^ ion at different concentrations. It can be seen that red channel had the highest sensitivity. Moreover, the results of Fig. 5e show that the nitrite ion with a concentration as low as 50 μM can be detected in artificial urine and the calculated LOD based on the results of the blue channel was 0.13 mM.

For the results that we reported up to this point, our paper-based sensors for cations, i.e., Na^+^, K^+^, and Ca^2+^, were quantified via a benchtop reader. For anions, i.e., Cl^−^, and ${\text{NO}}_{2}^{-}$, our paper-based sensors were imaged via a smartphone camera, and processed with a MATLAB script on PC, which can be easily translated to a smartphone application. As the next step, we replaced the benchtop reader with a smartphone-enabled platform (Fig. 1f) to measure the concentration of cations. In order to obtain higher sensitivity and lower LOD, we further optimized the concentration of fluorescent probes, and increased the concentration of fluorescent probes to 250 µM (last three rows in Table 1). Figure 6 shows the fluorescent intensity of the probes in the presence of different ion concentrations. Calibration parameters and LOD of Na^+^, K^+^, and Ca^2+^ in artificial urine are summarized in Table 1. Na^+^ quantification via the chosen fluorescent probe in this study was limited beyond 65 mM concentration of Na^+^. However, the presented smartphone-enabled setup would be still useful for lower range of Na^+^ concentrations, and could report binary results for Na^+^, such as “healthy: within physiological range” or “not healthy”, together with a specific concentration reported with a LOD of 1.26 mM (Table 1). For K^+^ and Ca^2+^ sensing in urine, our smartphone-enabled platform provided a robust correlation for both physiologically relevant range and higher concentrations. Overall, these results show that our smartphone-enabled platform is promising for a wide range of cation concentrations, especially for K^+^, and Ca^2+^, and for binary quantification of Na^+^.

## Conclusions

To address the emerging needs for measuring the concentration of electrolytes, such as Na^+^, K^+^, Ca^2+^, Cl^−^, and ${\text{NO}}_{2}^{-}$ ions in body fluid, especially for the people with high blood pressure issues and kidney diseases who need to regularly track their intake of sodium, potassium and calcium, we proposed a smartphone-enabled device for quantifying the concentration of these ions in urine. Using this method, we demonstrated the ability to use a miniaturized paper-based, smartphone-enabled device for quantification of electrolytes in artificial urine by fluorescent and colorimetric detection methods. By using this cost-effective device, daily quantification of ions in urine would be feasible, which can result in both monetary and time savings for people needing regular detection. The proposed device can be further utilized for diagnosing diseases such as hypertension, hyperkalemia or hypokalemia, kidney disease, cystic fibrosis, and urinary tract infection (UTI) by measuring the concentration of Na^+^, K^+^, Ca^2+^, Cl^−^, and ${\text{NO}}_{2}^{-}$ ion in urine.

## Supplementary information

Supplementary information.
