# Supplementary material for: Sensing of electrolytes in urine using a miniaturized paper-based device
Source: Sci Rep. 2020 Aug 12;10:13620. doi: 10.1038/s41598-020-70456-6 (PMC7423618; doi:10.1038/s41598-020-70456-6)
Supplement: Supplementary file 1 — Supplementary information. [file 41598_2020_70456_MOESM1_ESM.pdf]

SUPPLEMENTARY INFORMATION

Sensing of electrolytes in urine using a miniaturized paper-based device

Fariba Ghaderinezhad[1], Hatice Ceylan Koydemir[2,3,4], Derek Tseng[2,3,4], Doruk Karinca[5], Kyle Liang[5], Aydogan Ozcan[2,3,4,*], Savas Tasoglu [6,*]

[1] Department of Mechanical Engineering, University of Connecticut, Storrs, CT 06269, USA
[2] Electrical and Computer Engineering, University of California, Los Angeles, CA 90095, USA
[3] Bioengineering, University of California, Los Angeles, CA 90095, USA
[4] California NanoSystems Institute, University of California, Los Angeles, CA 90095, USA
[5] Computer Science, University of California, Los Angeles, CA 90095, USA
[6] Department of Mechanical Engineering, Koc University, Sariyer, Istanbul 34450, Turkey

* Authors to whom correspondence should be addressed. Emails: ozcan@ucla.edu and stasoglu@ku.edu.tr

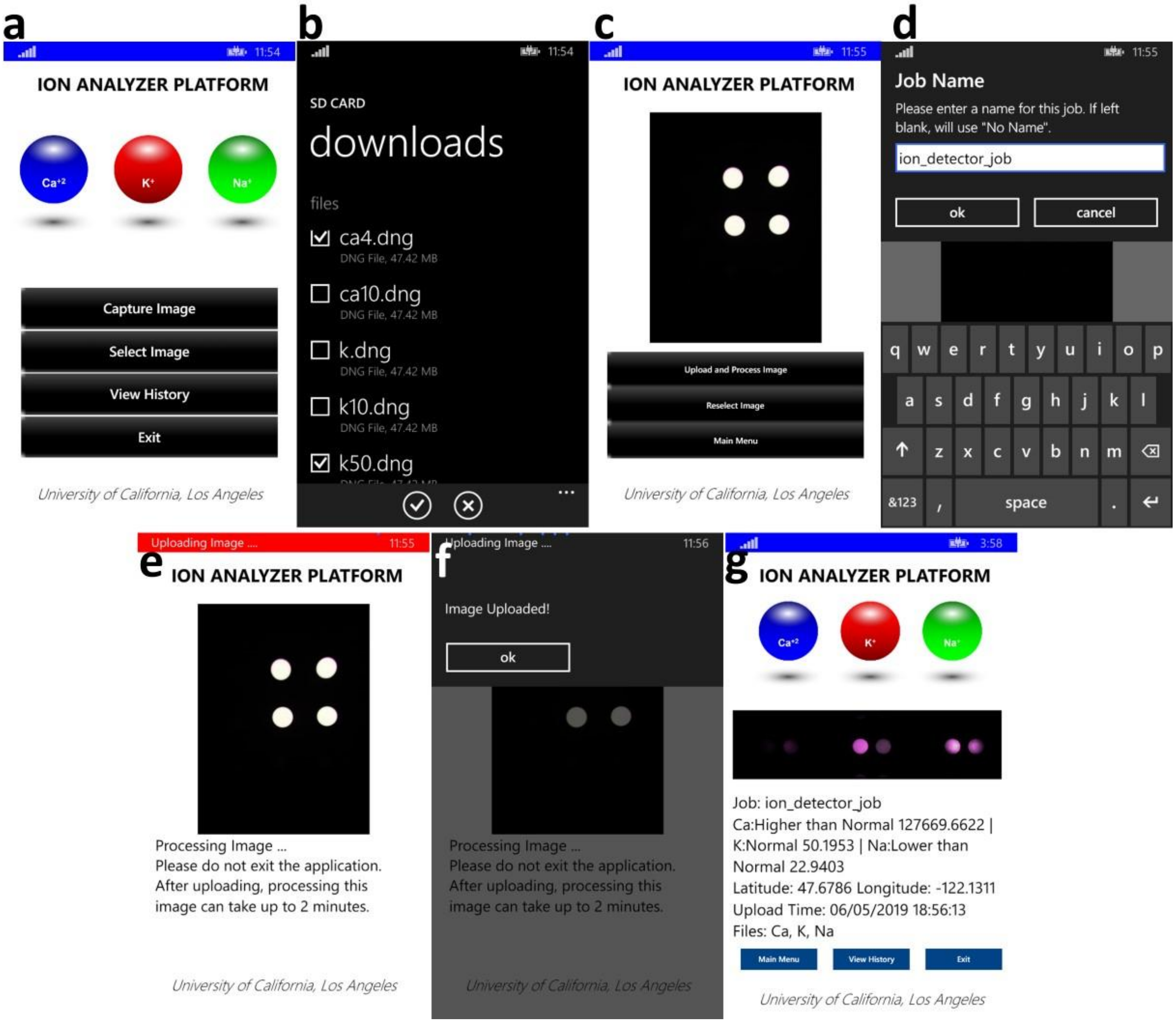

Figure S1. Screenshots of the custom-developed application running on our smartphone-based platform for measuring the concentrations of Na^{+}, K^{+}, and Ca^{2+} ions in urine samples.
